# Medical staff's cognition and experience of specialist nurse practice in critical care unit: a qualitative study

**DOI:** 10.3389/frhs.2025.1720425

**Published:** 2026-01-22

**Authors:** MengJuan Jing, XiaoJing Wei, ChunPeng Li, YuLin Xu, LiMing Li, Hao Li

**Affiliations:** 1 Department of Critical Care Medicine, Henan Provincial People’s Hospital, Zhengzhou Key Laboratory of Critical Care Nursing, Zhengzhou, China; 2 Department of Cardiovascular Medicine, The First Affiliated Hospital of Zhengzhou University, Zhengzhou, China

**Keywords:** advanced practice nurse, intensive care, nursing challenges, qualitative study, specialist nurse

## Abstract

**Background:**

Specialist nurses represent an important pathway through which registered nurses transition toward advanced practice nurse roles, and their development and clinical practice are essential for addressing contemporary healthcare challenges. However, in countries such as China, substantial gaps remain in the training and practical implementation of specialist nurses. Despite more than two decades of specialist nurse training in China, systemic issues such as unclear role definitions, insufficient designated positions, and limited decision-making authority continue to constrain their clinical practice.

**Objective:**

To investigate healthcare professionals' perceptions and experiences of specialist nurse practice in ICU to identify their roles, impacts, challenges, and suggestions for improvement.

**Setting:**

A 25-bed ICU in a tertiary hospital in central China has been conducting under the specialist nurse practice model for six months.

**Participants:**

A purposive sampling survey recruited 18 nurses (14 ICU registered nurses and 4 specialist nurses) and 3 ICU physicians, all with more than one year of ICU work experience.

**Methods:**

A qualitative descriptive design using semi-structured interviews was adopted.

**Findings:**

Three major themes emerged from the analysis: 1) being a versatile and core force; 2) leading nursing quality and professional transformation; and 3) facing challenges and pursing a path to growth.

**Conclusion:**

Specialist nurses play a pivotal role in ICU nursing, driving quality improvement and professional development. However, systemic barriers such as resource constraints and role ambiguity limit their full potential. Addressing these challenges through workflow optimization, clear role definition, and policy reforms is crucial for advancing specialist nurse practice globally.

## 1 Background

Advanced practice nurse (APN) is growing rapidly around the world as the increasing burden of disease care, shortage of health resources, and continuous deepening of nursing expertise (1). APN refers to a registered nurse who has acquired rich specialized knowledge, decision-making ability in complex problems, and clinical competencies through education (2). APN is advocated as an effective and efficient resource for addressing health care challenges (3–5).

APN demands a extensive knowledge, informed decision-making, and proficient skill, so a master's degree and an expanded scope of practice are considered essential components of the APNs (2). However, there are significant differences in nursing education in countries, some countries have an insufficient pool of master's and doctoral nursing professionals (6). The International Council of Nurses (ICN) notes that in countries where general nursing education is progressing and master's degree education is being considered for the development of APN, transition programs can be developed to prepare for the development of specialty nurses (SN) into APN roles (2). SN refer to registered nurses (RN) who have undergone training in a specific field, have the appropriate specialist nursing competence, and have passed the examination and obtained the specialist qualification certificate. Unlike APNs, SNs generally do not possess advanced clinical authority or master's-level preparation, although this may vary by region and individual educational background (2). Multiple studies have demonstrated that specialist nurse-led practice in specific clinical domains yields positive effects, improving patient outcomes and reducing readmission rates (7–9).

It is acknowledged that nursing higher education in China started late. Experienced nurses in specialty areas have not received a master's degree or higher education. Conversely, nurses who graduated with a master's degree lack clinical experience (10). Therefore the development of SN has become one of the main ways to explore the role of APNs in China. SN were first introduced in Hong Kong, China in the 1990s. The training of specialized critical care nurses was first conducted in mainland China in 2002, with the support of the Hong Kong Association of Critical Care Nurses. In 2007, the Government issued a Training Curriculum for Nurses in Specialized Nursing Areas to guide and regulate the training of nurses in five specialists, including intensive care, operative, emergency, organ transplantation and oncology (11). SN have flourished in China in the following years. At present, the training of SN is led by the Chinese Nursing Association or provincial nursing associations, with qualified teaching hospitals as the training bases. Those who have received training and passed the assessment will be awarded a certificate of SN by the organizer. However, there is still no unified organization to recertify the qualifications of SN.

SNs have been in development for more than two decades, yet, as in many countries, China is still working on “how to train” more outstanding nurse, while the importance of “how to use” has been widely overlooked (12). A study included 1,846 SNs showed that 73.02% of SN did not have a specific job position after they were certified, and the scope of practice for most SN remains virtually unchanged (22). Another study of 792 tertiary hospitals showed that only 38.0% of the hospitals had a remuneration system for SN (14). This indicates that the phenomenon of neglecting the management of SNs is very common. Indeed, a lacked system for the management and utilization of SN is an issue that severely limits the role and value of SN in clinical practice.

In order to promote the development of SN in China, we conduct a clinical practice of SN in ICU. This is an original study in which four specialized nurses (in wound care, nutrition, rehabilitation, and intravenous therapy) are assigned independent schedules, separate from the regular nursing shift rotations. In China, wound care, intravenous therapy, rehabilitation, and clinical nutrition are officially recognized Specialist Nurse categories within national and provincial certification systems. In our clinical practice, their role is to provide clinical care related to their specialty for all patients in the intensive care unit, rather than being limited to only 2–3 patients. The purpose of this study is to explore medical staff's cognition and experience of the SN practice in ICU, which helps to understand the benefits and obstacles of SN practice. We believe that this study can provide valuable experience in SN practice for some countries that are late in the development of nursing education.

## 2 Methods

### 2.1 Design

We adopted a qualitative descriptive design, which is well suited for capturing healthcare professionals' experiences and perspectives regarding SN practice and for generating a structured, practice-proximal understanding of the phenomenon. Data were analyzed using reflexive thematic analysis following Braun and Clarke's six-phase framework. The analytic process involved: (1) familiarization through repeated reading of transcripts and listening to audio recordings; (2) inductive, line-by-line coding with a primarily semantic orientation; (3) generating initial themes by clustering conceptually related codes; (4) reviewing and refining themes to ensure internal coherence and clear boundaries between themes; (5) defining and naming themes; and (6) producing the final report through iterative movement between data, codes, and candidate themes. The overall analytic approach was inductive and data-driven, without imposing *a priori* theoretical categories. Reporting adhered to the Consolidated Criteria for Reporting Qualitative Research (COREQ) (15).

### 2.2 Study setting and recruitment

When this study began, the SN practice model had been in place for six months in a 25-bed ICU staffed by 70 RNs and 10 physicians in a tertiary hospital in central China. The unit typically operated with a nurse-to-patient ratio of about 1:3. In this study, RNs refer to general ICU nurses responsible for routine patient care, whereas SNs are ICU RNs who have completed formal specialty training and certification in areas such as wound care, nutrition, rehabilitation, or intravenous therapy. The model included four full-time SNs working an 8-hour weekday schedule (08:00–12:00 and 14:00–18:00) independent of rotation shifts, with their responsibilities summarized as shown in Table 1. Consults could be initiated by bedside nurses or physicians, and SNs documented their assessments and communicated recommendations to the multidisciplinary team. SNs reported to the ICU head nurse and participated in weekly quality meetings. Their workload included specialty consults, reassessments, catheter and skin audits, wound rounds, and updates to nutrition plans.

**Table 1:** The responsibilities of a SN.

Wound care SN	Nutrition SN	Rehabilitation SN	Intravenous therapy SN
Participate in the morning meeting and doctor's rounds, familiarize yourself with the patient's medical history, present illness, positive signs, examination and lab results, etc., and record the patient's diagnosis, treatment, prognosis, and current major nursing problems			
Evaluate the implementation of the patient's skin care plan and assess the effectiveness of nursing care	Evaluate the implementation of the patient's nutritional support plan, assess the effectiveness of its execution, and adjust the plan as needed	Evaluate the patient's physical function and develop personalized rehabilitation measures	Evaluate the use, maintenance, and complications of the patient's vascular access
Document each patient's specialty-related nursing issues and challenging nursing points for discussion			
For complex cases, review the literature, participate in multidisciplinary team meetings, provide insights from your professional field, and collaboratively develop nursing plans, goals, and interventions			
Record the field-related nursing measures at the foot of the bed and inform the responsible nurse			
Manage pressure injuries, incontinent dermatitis, various stomas, chronic non-healing wounds, etc. If necessary, contact the hospital's stoma and wound specialist outpatient clinic for consultation	Manage feeding intolerance, gastrointestinal dysfunction, complex malnutrition, refractory hyperglycemia, etc. If necessary, contact the hospital's nutrition department for consultation	Conduct rehabilitation and contact respiratory therapists and hospital rehabilitation departments for consultation if necessary	Based on the patient's needs, select the appropriate intravenous catheter to establish access, and contact the hospital's intravenous therapy team for consultation if necessary
Collect clinical data for difficult, rare, and typical cases to facilitate clinical teaching			
Check whether the responsible nurse's nursing record accurately describes the skin condition, and whether the wound record and skin informed consent form have been established	Check whether the responsible nurse's nutritional support method is appropriate, whether the implementation of nutritional support follows proper procedures, and whether the NRS 2002 nutritional screening form is correctly completed	Check whether the responsible nurse has recorded the rehabilitation treatment process, including nursing interventions, treatment responses, and patient feedback.	Check whether the medical and nursing procedures for CVC and PICC insertion and maintenance are performed according to standard protocols
Assist in completing other tasks within the specialty field			
Identify clinical issues, collect relevant data, and engage in scientific research as well as the development of new business and technologies			

Over the two-month period from June to July 2023, we used purposive sampling with the intention of achieving maximum variation. In addition to diversity in age and years of work experience, we also sought variation across professional role (ICU RNs, SNs, and ICU physicians) and shift pattern (day/night rotation vs. weekday schedule). This approach ensured representation of staff occupying different positions within the specialist nurse practice model. Having more than one year of ICU work experience was the inclusion criterion for this study. Because the number of SNs and ICU physicians in our unit was limited, these two groups were not recruited based on the saturation principle; instead, all available individuals were invited, resulting in the inclusion of four SNs and three physicians. In contrast, the number of ICU RNs was determined according to the information saturation rule. Recruitment continued until no new codes emerged across three consecutive interviews, followed by two additional confirming interviews. A total of 14 ICU RNs were ultimately included. During the recruitment process, 27 invitations were sent out, of which 2 physicians and 4 ICU RNs declined participation.

### 2.3 Data collection

A semi-structured, face-to-face approach was used to conduct the interviews. All interviews were conducted by the first author in a private nurses' lounge to ensure a quiet and uninterrupted environment, while the corresponding author documented non-verbal cues during the process. The first author is a female ICU nurse with a master's degree and formal training in qualitative research. Although familiar with SN practice, the interviewer did not hold any supervisory role over participants, thereby reducing potential hierarchical influence. Prior to data collection, participants were informed of the study purpose and procedures, and written consent for audio recording was obtained. Demographic information, including age, educational level, and years of clinical experience, was collected before the interviews.

Interviews were guided by a semi-structured interview guide designed to explore participants' perspectives on SN practice in the ICU. Key questions included: What do you perceive as the unique value of Specialist Nurses within the ICU team? How does the presence of SNs influence your daily work? What challenges do SNs face in clinical practice? Is there anything else you would like to add regarding your experience with SN practice? All interviews were conducted in Mandarin, audio-recorded, and accompanied by contemporaneous notes capturing non-verbal expressions and body language. Only the interviewer and participant were present during the interviews. Interview duration ranged from 39 to 61 min. Interviews were transcribed verbatim, and transcripts were returned to participants for verification to enhance credibility.

### 2.4 Data analysis

Qualitative data analysis was led by the corresponding author and a co-author. The audio content of the interviews was transcribed verbatim using voice transcription software (16). Researchers then listened to the recordings while reviewing and correcting the transcriptions, adding any non-verbal information noted during the interviews. Thereafter, data were analyzed using reflexive thematic analysis following Braun and Clarke's six-phase framework. Two researchers repeatedly listened to and read through the interview content to become familiar with the data, identifying and extracting meaningful content. A manual coding method was used to code the content, with similar or related codes categorized into themes and sub-themes, ultimately determining the main themes. The analysis was primarily inductive, with all codes and themes generated from the data rather than guided by pre-existing theoretical frameworks. Our coding focused mainly on the semantic level, attending to participants' explicit meanings, while remaining open to deeper interpretive patterns when warranted. Data analysis was conducted independently by two researchers, who recorded and discussed any disputed sections. If necessary, a third researcher was consulted to reach a consensus.

### 2.5 Ethical considerations

The study was approved by ethics committee of Hospital (approval number: 20230330). Participants were provided with informed consent forms, which included methods of signing informed consent, as well as principles of voluntariness and confidentiality during the interview process, data handling, and the research reports.

### 2.6 Rigour and reflexivity

Rigour was ensured through strategies addressing credibility, dependability, confirmability, transferability, and reflexivity. Credibility was enhanced through a literature-informed semi-structured interview guide, audio-recorded interviews, detailed field notes, and independent analysis by two researchers. Dependability was supported by a systematic and transparent analytic process, with an audit trail documenting decisions throughout data collection and analysis. Confirmability was strengthened through verbatim transcription, cross-checking against audio recordings, and reflexive memo writing. Transferability was facilitated by rich descriptions of the ICU setting, the SN practice model, and participant characteristics.

Reflexivity was integral to the reflexive thematic analysis and contributed to rigour and analytic sincerity. The research team acknowledged the potential influence of professional backgrounds; in particular, the first author is an ICU nurse familiar with SN practice, which may predispose positive assumptions. To mitigate this, the interviewer held no supervisory role over participants, and reflexive strategies—including bracketing prior to interviews and coding, memo writing, and peer debriefing—were used throughout the study. Reporting followed the COREQ checklist (15).

## 3 Findings

Participants in this study included 14 ICU RNs, 4 SNs and 3 ICU physicians. The mean age of these medical staff was 33 years (25–43 years). All the nurses have a bachelor's degree, and all three ICU physicians hold a master's degree. Table 2 shows the detailed demographic characteristics of these participants.

**Table 2:** Demographic characteristics of participants.

Number	Role	Genders	Age	Education	Years of ICU experience
SN1	Nutrition SN	female	34	Bachelor	12
SN2	Rehabilitation SN	female	43	Bachelor	18
SN3	Wound care SN	male	37	Bachelor	15
SN4	Intravenous therapy SN	female	37	Bachelor	14
RN1	ICU RN	female	36	Bachelor	13
RN2	ICU RN	female	29	Bachelor	6
RN3	ICU RN	female	34	Bachelor	12
RN4	ICU RN	male	25	Bachelor	2
RN5	ICU RN	female	41	Bachelor	18
RN6	ICU RN	male	34	Bachelor	9
RN7	ICU RN	female	32	Bachelor	12
RN8	ICU RN	female	26	Bachelor	4
RN9	ICU RN	male	30	Bachelor	6
RN10	ICU RN	female	29	Bachelor	6
RN11	ICU RN	female	36	Bachelor	12
RN12	ICU RN	female	37	Bachelor	13
RN13	ICU RN	male	25	Bachelor	2
RN14	ICU RN	female	32	Bachelor	12
D1	ICU physician	male	34	Master	7
D2	ICU physician	male	34	Master	7
D3	ICU physician	female	36	Master	7

The following three themes and related categories were identified: (1) being a versatile and core force; (2) leading nursing quality and professional transformation; and (3) facing challenges and pursing a path to growth.

### 3.1 Theme 1—being a versatile and core force

#### 3.1.1 Direct caregiver

Almost all of nurses indicated that specialty care competencies are the foundation of the SN. Clinical practice are core to competency as a SN.

SNs are proficient in the knowledge and skills of their specialized fields and are capable of providing care for critically ill or rare patients.

(RN1)

Their professional skills are truly outstanding! There was a patient with a biliary fistula, and the drainage fluid leakage was very severe. The SN provided excellent care for the stoma wound, which completely exceeded my expectations.

(SN4)

Through systematic assessment and precise nursing interventions, SNs ensure that patients receive the best possible treatment outcomes.

(SN3)

#### 3.1.2 Communicator and coordinator

The medical staff indicated that the SN assumed the role of communicator and facilitator, coordinating and managing conflicts and conveying information efficiently and accurately.

There is a difference in the understanding of medical orders between doctors and nurses. People tend to consider issues from their own perspective. We understand both the nurses' feelings and the physicians' intentions, and act as intermediaries to coordinate and resolve this conflict.

(SN4)

I think it promotes the physician-nurse relationship to become better, the SN can understand why you are asking for that, and after giving a medical order or expressing a goal, they will try very hard to understand you and work hard to reach your goal.

(D3)

When encountering a family member who is difficult to communicate with, perhaps the SN is a more appropriate communicator compared to the RN. As the SN is able to give a more professional explanation, this can reduce nurse-patient conflicts.

(RN14)

#### 3.1.3 Manager and supervisor

The SN plays a leadership role in quality management of nursing and physician operations and leads quality improvement projects for system promotion.

For SN, I have summarized five key terms: discover, address, supervise, resolve, and improve. In their work, they are able to identify more detailed issues, such as low pressure tube cuffs. They can directly point out the problem and promote its correction.

(RN2, RN4)

In fact, there are some tasks that we are also aware of, but we tend to forget when we get busy. They are able to notice these details and help us improve the shortcomings in our clinical work.

(RN7)

We carry out some quality control tasks, such as monitoring the compliance rates of hand hygiene among medical staff, bed elevation, and catheter maintenance. We provide monthly feedback to encourage everyone to follow the protocols and standards.

(SN2)

#### 3.1.4 Consultant and educator

SN can provide consultation to both family members and nurses, while also assisting the multidisciplinary team in educational training.

Some issues are more likely to be consulted with SN rather than physicians.

(RN5)

Teaching is an important part of the SN's work. When I perform a PICC catheter insertion, a large group of learners shows interest, including not only nurses but also resident doctors who come to observe.

(SN4)

#### 3.1.5 Researcher

SN should possess research abilities, such as identifying clinical problems, searching for evidence, and translating it into practice to reduce the gap between practice and evidence. Using scientific methods to conduct nursing research and promote academic exchange is also crucial.

Research has always been my weak point, but now I can easily browse through literature and independently submit conference papers. I have reviewed a lot of literature because I need to use evidence, guidelines, and consensus to guide clinical work, rather than relying solely on clinical experience.

(SN4)

SNs are more likely to encounter complex cases. They have an advantage in data collection and, therefore, greater opportunities to publish articles and share their experiences, making it easier to achieve success in research.

(RN10)

### 3.2 Theme 2—leading nursing quality and professional transformation

#### 3.2.1 Improved quality of care

SN are able to provide personalized and continuous care for ICU patients, improving the quality of nursing.

The RNs for a patient is not fixed, leading to a lack of dynamic follow-up. However, SNs continuously focus on key patients and provide continuous care.

(RN1)

Professionals do professional things. Now, the management of patients' catheters and skin-related complications has become more standardized, which certainly benefits the patients. The refined and personalized care provided by SNs is much better than RNs.

(RN3, RN5)

#### 3.2.2 Increasing professional value

The interviewees believe that SN can increase nurses' sense of professional achievement and value.

There was a patient with gastrointestinal bleeding, and I suggested some nutritional advice to the doctor, which was accepted. In the end, the patient recovered well. I was involved in the entire nutritional process for this patient, which gave me a great sense of accomplishment. Now, when there are nutrition-related issues, everyone messages me privately, indicating that I am still recognized.

(SN1)

The physician said 60% of the credit for the patient's recovery should go to me. I was very happy to hear this, as it made me feel the value of my work.

(SN2)

There was a physician who rotated to another department, and when he had a patient with a skin issue, he still called me for a consultation regarding the wound care. My expertise was recognized, and this recognition strengthened my confidence in continuing to persevere.

(SN3)

#### 3.2.3 Motivating nurses to improve

The practice of SN have raised higher educational demands, promoting the enhancement of their professional competencies. At the same time, they help RNs clarify their career paths and inspire them to join the ranks of SN.

I feel immense pressure now, fearing that I might not be able to answer others' questions. This pushes me to learn a lot more because otherwise, how could I be worthy of the SN position?

(SN4)

Although I have received training in relevant knowledge, what I've learned is far from enough. Specialized concepts are constantly evolving, and I continuously improve my knowledge and skills through reviewing literature.

(SN3)

I want to become an SN, which drives me to explore and delve into a specific field. I see this as a source of motivation.

(RN1)

### 3.3 Theme 3—facing challenges and pursing a path to growth

#### 3.3.1 Human resources challenges

Medical staff widely reported that the lack of human resources is the most significant challenge in SN practice.

When several nurses are assigned to independent schedules and no longer participate in regular day and night shift rotation, the staffing of RNs becomes somewhat insufficient.

(RN11)

There are a large number of patients, and several highly experienced nurses have been reassigned to work as SN. As a result, the number of RNs is severely insufficient.

(D2)

#### 3.3.2 Blurred workflow and responsibilities

The specific job content and workflows of SN still need further clarification and definition.

As a RNs, of course, I hope SNs can do more to share my workload. Some of their tasks are ambiguous, with no clear boundaries, so their responsibilities should be clearly defined.

(RN1)

I don't know the specifics of their work. I feel their roles are very flexible and rely heavily on self-motivation. Their job responsibilities need to be more detailed.

(RN8)

#### 3.3.3 Decision-making power dilemma

Nurses lack prescribing authority, leading to treatment delays and decision-making conflicts.

Any decision made by SNs must be communicated with doctors in advance, even in areas they excel at.

(RN6)

The assessment of indications and insertion of nasojejunal tubes are all carried out by SNs. However, they still have to wait for a doctor's order before proceeding. Why not grant prescribing authority to specialist nurses?

(RN8)

## 4 Discussion

In countries where nursing education is underdeveloped, SN are the primary pathway for RNs to transition to APN (17). Therefore, the development of SNs is considered crucial. The practical experience of SNs is extremely valuable; however, relevant research reports are unfortunately scarce worldwide. We conducted a six-month SN practice in the ICU and explored the experiences of medical staff regarding SN practice through qualitative research methods. Our study aims to assist countries that are planning or currently exploring SN clinical practice. Three main themes were identified: being a versatile and core force, leading nursing quality and professional transformation, and facing challenges and pursing a path to growth.

Our healthcare professionals reported the multiple roles and outstanding contributions of SN in ICU practice. First of all, direct professional practice ability is crucial for SNs, as solid clinical practice skills enable them to excel in other areas. Unlike APN, who are required to provide comprehensive care and make accurate diagnoses and sound decisions for complex healthcare issues, SNs place more emphasis on excellence in specific areas of practice. Furthermore, communication and coordination, as well as management and supervision, are strong manifestations of leadership in SNs (18). Each member of the ICU team brings unique clinical expertise and insights to patient care, with nurses serving as key contributors in coordinating and integrating medical resources (19). Nurses with leadership skills not only effectively guide patients but also lead other caregivers in joint participation in nursing practice to achieve care goals (20). Additionally, providing consultation and education is a core competency of SNs. Not surprisingly, SNs, who possess rich experience and expertise in a specific field, easily provide consultation and guidance to patients, as well as education and training to team members. This is not only their responsibility but also their strength. Through effective communication with patients and the team, SNs can translate complex medical knowledge into easily understandable information, helping patients better manage their health issues and enhancing the overall capability of the team. This interaction and knowledge-sharing promote more efficient nursing practices and better patient outcomes (21). Research capability is another important competency mentioned by the interviewees. SNs are not only executors of nursing care but also drivers of nursing science and practice innovation (22). For SNs, research is not only about applying scientific research methods to explore new approaches and models but, more importantly, about participating in and leading the implementation of evidence-based practice (23, 24). However, as in many other countries, research capability remains a shortcoming for SNs (25).

Leading nursing quality and professional transformation was the second major component reported by interviewees. In this model, more patients are able to directly benefit from the expertise of SNs. Some studies have shown that missed nursing care are common in clinical settings and have become a global concern (26, 27). This issue may be more pronounced in China, where research indicates that 91.8% to 95.1% of ICU nurses have experienced missed nursing care (28, 29). The causes of this phenomenon include not only a lack of human resources and excessive nursing workload but also insufficient awareness and lack of attention. SNs' clinical practice effectively addresses these issues. Our study shows that SNs, who possess more knowledge and experience in their field, help reduce missed nursing care caused by inexperience or knowledge gaps among RNs by participating in direct care and quality management.

On the other hand, the respondents reported that SN led professional transformation, and more RNs felt a greater sense of professional value. Historically, a lack of social recognition and limited career development opportunities have been considered the main reasons for nurses' low sense of professional value and one of the causes of nurse turnover (30, 31). The establishment of SN practice brings more challenges, promoting nurses to accumulate richer professional knowledge and skills. At the same time, greater autonomy allows nurses to exert a greater influence within the healthcare team. Furthermore, specialized nursing services not only gain the respect of colleagues but also provide patients with a better healthcare experience, enhancing societal recognition of the nursing profession. Overall, the practice of SN significantly enhances nurses' sense of professional value by improving their professional capabilities, increasing their career influence, and gaining social recognition.

This benefits both the individual nurse's career development and helps stabilize the nursing workforce. The practice of SN not only enhances the individual nurse's sense of professional value but also has a positive motivating effect on the entire nursing community. By continuously improving their professional skills and knowledge, SNs can play a larger role in clinical work, improve the quality of care, and help patients achieve better rehabilitation outcomes. These achievements not only boost the confidence and sense of accomplishment of SNs but also serve as role models for other nurses. When the nursing community sees SNs gaining high recognition from patients and respect from society through their specialized services, it often motivates them to pursue professional development, thereby increasing their sense of professional identity and pride.

While SN practice brings significant benefits, it also faces a series of dilemmas and challenges, which are an inevitable part of developing specialist nursing. These challenges mainly focus on human resources, unclear workflows and responsibilities, and decision-making authority dilemmas. The shortage of human resources is the primary challenge and one of the key factors hindering the development of SN practice. Generally, developing countries have relatively scarce healthcare resources, resulting in a typically lower nurse-to-patient ratio. According to recommendations from the European Society of Intensive Care Medicine, the ICU nurse-to-patient ratio is typically 1:1–1:2 (32), and in the United States, it is about 1:2 (33). However, in China, the ratio ranges from 1:2 to 1:3 (34), meaning that ICU nurses in China are responsible for more nursing tasks with the same number of patients. A study on the current status of SN work in Chinese ICUs indicates that insufficient nursing human resources make it difficult for SNs to transition from their original positions, meaning that full-time SNs are very rare (35). In our study, all four SNs worked full-time, transitioning from day and night shifts to regular weekday shifts, which resulted in a reduction in the number of RNs. Unsurprisingly, most respondents reported an increased workload. When the four experienced nurses joined the SN practice, RNs had to sacrifice some of their rest time to cope with the reduced human resources. We believe this is one of the most common challenges in healthcare institutions, but it does not mean that there is no solution. Addressing this issue requires a multi-faceted approach, such as optimizing nursing workflows and reducing unnecessary task duplication through more refined nursing management. Additionally, introducing more intelligent devices could help alleviate the nurses’ workload (36).

Another issue raised by the respondents is the unclear responsibilities and workflows of SNs, which not only affects the efficiency of SNs but also creates pressure for RNs. We acknowledge that this is an exploratory study, and the practice of ICU SNs in China is indeed a relatively new field, lacking mature theoretical frameworks and practical experience. In this context, the lack of clarity in the responsibilities and workflows of SNs is expected. To clarify the roles of SNs, it is essential to document the work content. Although we have developed a duty manual that includes work regulations and job descriptions, this is far from sufficient. Work processes and assessment methods could also be clarified to ensure that their tasks are not only feasible but also quantifiable and trackable. Additionally, participants indicated that establishing clear boundaries of responsibility may be equally important. Defining the division of responsibilities between SNs and RNs may help avoid ambiguous job scopes. This not only helps SNs work in an organized manner but also helps RNs understand the SNs’ tasks, reducing differences in expectations between them. Furthermore, establishing a feedback mechanism could help ensure that SNs receive ongoing feedback and supervision from RNs. This will not only help SNs improve work efficiency but also enhance the satisfaction of RNs.

The decision-making authority dilemma is the last challenge mentioned by the respondents in this study. In China's healthcare system, physicians are often at the core of the decision-making chain, while nurses are more execution-oriented than decision-makers (37, 38). In our practice, even though SNs have a certain level of influence within the healthcare team, relevant laws, regulations, and hospital policies have not explicitly granted SNs the authority to make medical decisions, such as adjusting medications or inserting or removing feeding tubes. This creates limitations where SNs may face the situation of “wanting to act but unable to,” which impacts their professional satisfaction. On the other hand, nursing decisions often need to be made quickly and flexibly. Without independent decision-making authority, it may affect the timeliness and quality of patient care. Although resolving the dilemma of nursing prescribing authority primarily depends on improvements at the institutional and policy levels, managers could also take measures to address this issue. First, through training and continuing education, SNs' professional abilities, particularly decision-making-related knowledge and skills (such as nursing assessments, patient condition evaluations, etc.), should be strengthened. Additionally, enhancing communication and collaboration between physicians and SNs may could help build trust in SNs' professional competence, making physicians more willing to accept their suggestions and judgments. Furthermore, within multidisciplinary teams, the professional role and decision-making authority of SNs need to be clearly defined to enable they have a voice in team decision-making. In conclusion, the decision-making authority dilemma is a key challenge in the development of SN roles, particularly in China, where this field is still in the exploratory phase. We believe that by improving SNs' abilities, establishing trust between physicians and nurses, and optimizing multidisciplinary team collaboration, this dilemma may could gradually be resolved, paving the way for the development of SNs.

## 5 Limitations

As this is a pioneering study, it is based on the practical experience of a single ICU in a tertiary general hospital located in central China. The sample source is singular, and the findings primarily reflect the organizational, cultural, and clinical context of this specific center, rather than comprehensively representing practices in other regions, hospitals of different sizes, or various types of ICUs. In addition, this study adopted a qualitative design focusing on healthcare professionals’ perceptions and experiences of SN practices. As such, the findings do not provide objective outcome measures and do not allow for causal inference regarding the effects of SN practices on nursing quality, team collaboration, or clinical outcomes. The results should therefore be interpreted as exploratory and hypothesis-generating, rather than confirmatory. Furthermore, the six-month practice period is relatively short and may not be sufficient to fully capture the long-term impact of SN practices on team dynamics and professional role development. In the context of pioneering implementation, practice effects may require a longer period to stabilize and fully manifest; therefore, the findings may predominantly reflect an early exploratory phase. Moreover, only four SNs were involved in the practice. Individual differences, such as personal competencies, prior experiences, leadership styles, and communication skills, may have influenced participants' perceptions and contributed to variability in the findings. Finally, this study focused on the perspectives of healthcare professionals and did not incorporate the views of patients and families. As a result, the direct impact of SN practices on patient satisfaction, communication experiences, and patient-centered outcomes may not be fully captured, which limits the comprehensiveness of the findings. Future research may benefit from including multiple departments and institutions to enhance transferability, conducting longer-term follow-up to examine the sustained effects of SN practices, and integrating patient and family perspectives or quantitative outcome measures to more comprehensively evaluate the role and impact of SN practices.

## 6 Conclusions

This study explored healthcare professionals' perceptions and experiences of SN practices in the ICU, providing insights into how the roles, value, and challenges of SNs are understood in clinical practice. The findings suggest that healthcare professionals perceive SNs as fulfilling multifaceted roles in the ICU, including direct care providers, communicators and coordinators, managers and supervisors, as well as educators and researchers, highlighting their perceived importance within the clinical team. Participants reported that SN practices were associated with perceived benefits, such as enhanced nursing quality, strengthened perceptions of team value, and increased motivation among RNs to pursue professional development. At the same time, healthcare professionals also described several challenges, including strained nursing resources, unclear role boundaries and workflows, and limited decision-making authority, which were perceived as constraining the full enactment of the SN role. Overall, this study offers exploratory insights into healthcare professionals' views on SN practices in the ICU context. These findings may serve as a contextual reference for future research and discussions on the development and optimization of SN roles, rather than as evidence of causal effects or effectiveness.

## Data Availability

The original contributions presented in the study are included in the article/Supplementary Material, further inquiries can be directed to the corresponding author.
